# Information Density and the Extraposition of German Relative Clauses

**DOI:** 10.3389/fpsyg.2021.650969

**Published:** 2021-11-26

**Authors:** Sophia Voigtmann, Augustin Speyer

**Affiliations:** ^1^Department of Modern German Linguistics, Saarland University, Saarbrücken, Germany; ^2^Collaborative Research Center on Information Density and Linguistic Encoding, Saarland University, Saarbrücken, Germany

**Keywords:** information density, Early New High German, relative clauses, extraposition, corpus linguistics

## Abstract

This paper aims to find a correlation between Information Density (ID) and extraposition of Relative Clauses (RC) in Early New High German. Since surprisal is connected to perceiving difficulties, the impact on the working memory is lower for frequent combinations with low surprisal-values than it is for rare combinations with higher surprisal-values. To improve text comprehension, producers therefore distribute information as evenly as possible across a discourse. Extraposed RC are expected to have a higher surprisal-value than embedded RC. We intend to find evidence for this idea in RC taken from scientific texts from the 17th to 19th century. We built a corpus of tokenized, lemmatized and normalized papers about medicine from the 17th and 19th century, manually determined the RC-variants and calculated a skipgram-Language Model to compute the 2-Skip-bigram surprisal of every word of the relevant sentences. A logistic regression over the summed up surprisal values shows a significant result, which indicates a correlation between surprisal values and extraposition. So, for these periods it can be said that RC are more likely to be extraposed when they have a high total surprisal value. The influence of surprisal values also seems to be stable across time. The comparison of the analyzed language periods shows no significant change.

## Introduction

Attributive Relative Clauses (RC) provide more information about their head noun in their matrix clause. The head noun is taken up by the relative pronoun, the first word of the RC itself. One characteristic of German RC is that they can be placed adjacent to (1) or separated from their head noun (2).

When RC is separated from their head nouns, they are mostly placed in the Postfield (PoF), a position that is not usually mandatory. So, the question arises why RC can be frequently found there.

**Table:** 

**Prefield**	**LSB**	**Middle field**	**RSB**	**Postfield^1^ (PoF)**
1) Peter	Hat	Maria das *Buch*, das sie dringend braucht,	gegeben.	
Peter	Has	Maria the *book* that she urgently needs	given.	
2) Peter	Hat	Maria *das Buch*	gegeben	das sie dringend braucht.
Peter	Has	Maria *the book*	given	that she urgently needs.
“Peter has given Maria a book that she needs urgently.”				

Explanations for the phenomenon of RC extraposition^2^ vary between the length of the RC, the distance between PoF and RC head noun, the RC type, which can be divided into restrictive and non-restrictive RC, and the phenomenon called information disentanglement [“Informationsentflechtung” Zifonun et al. (1997, p. 1,669)]. This study examines the correlation between information and the position of relative clauses. There has been much research [Vinckel-Roisin (2015); Poschmann and Wagner (2016), among others] which links RC extraposition with information status or focus [both are often understood according to Chafe (1976); Prince (1981); Krifka (2007), among others].

For the purpose of this study, we, however, use the term information in reference to the Information Density (ID) of Shannon (1948) and define it as the “amount of information per unit comprising the utterance” (Levy and Jaeger, 2007, p. 1). Information is the likelihood of the occurrence of a word given a context of *n* words in terms of ID. Words that are frequent in a certain context have lower surprisal values than words that rarely occur in that context. Surprisal values correlate with perceiving and production difficulties [Hale (2001); Jaeger (2010), among others]. So, the impact of words with a high surprisal value on the working memory is higher than the influence of words with a low surprisal value. Therefore, speakers tend to distribute information as evenly as possible across clauses and discourses. Aylett and Turk (2004) found these effects in spoken languages. Levy and Jaeger (2007) extended their hypothesis for more contexts, like syntax, and formulated this principle in their “Uniform Information Density Hypothesis (UID).”

Information Density is well-established for measuring cognitive load and has already been used to explain RC extraposition in English with experiments and corpus studies [Francis and Michaelis (2012, 2014, 2017); Levy et al. (2012), among others]. Nonetheless, it has been rarely used for other languages such as, for instance, German (e.g., Voigtmann and Speyer, forthcoming). It is possible to connect all explanations for RC extraposition in German to the establishment of successful communication and the prevention of perceived difficulties. Only a few studies, however, immediately correlate perceiving difficulties with RC extraposition [e.g., Hawkins (1994) or Gibson (1998); Uszkoreit et al. (1998) for modern German] or test this correlation using ID (Voigtmann and Speyer, forthcoming; Speyer and Lemke, 2017). In this study, we apply the principle of establishment of successful communication, the main goal pursued by Shannon (1948), to the explanation of RC extraposition. The principles of ID are considered to be universal and testable on corpus data and are thus applied to historical data where RC extraposition, in general, is still under-researched. We aim to fill that gap.

In this study, we pose and discuss two hypotheses. First, regarding the extraposition of RC, we claim that the variability of RC positions is connected to perceiving difficulties that are caused by high surprisal values. Following Hawkins (1994) and Gibson (1998), the RSB marks the end of a clause. Processing capacities are free again so that RC with higher surprisal values are placed there without causing information loss. If RC with high surprisal values would be placed adjacent to their head noun between the sentence brackets, their processing could strain the processing capacities too much and information loss would happen. So, our first hypothesis is the following:

*(H1) Higher surprisal values in RC favor their extraposition*.

Second, we take the diachronic perspective of our corpus into account. We conducted a corpus study for Early New High German (ENHG)^3^ and early Modern German medical texts to test this hypothesis and provide information about an earlier stage of German. Due to few scientific texts in German in the seventeenth century, as scientific writing in Germany was done in Latin before that point, the effect might be different for the seventeenth than the nineteenth century. While the seventeenth century authors might only have a few scientific texts as a model, for nineteenth century authors, scientific articles written in their native language were already common. Developments in style and commonness of writing in native language of an individual instead of a Lingua Franca are taken into account by dividing the timespan into different parts. As the main goal of all authors in each time span is, however, still to ensure successful communication by means of their texts, we propose in our second hypothesis:

*(H2) The correlation between the extraposition of RC and their surprisal values is consistent over the centuries*.

We divided this time span of 250 years into periods of 50 years to be able to account for a change. Note that the New High German period (from around 1650 to 1900) is not subdivided like former language periods. Research concentrating on German in the eighteenth or nineteenth century does not base the division of the timespan on intra-linguistic criteria but takes century borders. For our subperiods, we used a smaller time span of only 50 years and understand this as an exploratory approach.

In this study, we try to find evidence for both hypotheses. Furthermore, we check whether information density is a better predictor for extraposition than restrictiveness and length because all factors factor frequently mentioned in literature are connected to perceiving difficulties. For a complete picture, we first present a more detailed description of RC and RC extraposition along with that of the ID of Shannon (1948) and the principles mentioned above (Section Theoretical Background). Then, we describe our corpus and method (Section Corpus and Method) before presenting the results (Section Results: Information Density and Length). The study closes with a discussion about the results (Section Discussion) and a conclusion (Section Conclusion).

## Theoretical Background

In the following section, we present the kind of RC used for this investigation and reasons for extraposition which includes restrictiveness, length, and information management for German RC. As far as possible we include research about ENHG but as this period is highly underrepresented in research, synchronic research will be included as well as an approach to Modern German standards.

The second part of this section gives an overview of Information Density, its usage, and some of its advantages. We concentrate mostly on the study of Shannon (1948) itself and only include some more recent research where it is relevant for our hypotheses. The main goal is to show how information is defined and how ID correlates with processing difficulties. For more details and mathematical evidence for the way ID is calculated, please see Shannon (1948) or Levy (2008), among others.

The connection between RC extraposition and ID will be drawn in section “Methodological Considerations About RC Extraposition and ID” because it also concerns the predictors used in our model and is, therefore, more suitable there.

### Relative Clauses

As mentioned in the introduction, RC are subordinate clauses. Besides bound or attributive RC (example 1, 2), on which this paper focuses, there are also free and continuous RC. They are excluded from this investigation either because they do not have an antecedent that is present in the sentence (free RC) or take the whole sentence as an antecedent and therefore can only be placed in the right periphery [continuous RC, for more information, refer to Gallmann (2005)].

Our definition of bound RC follows Hentschel and Weydt (2003), who define RC as clauses that apply attributively to their antecedent and which are introduced by the relative pronouns “der/die/das” or “welcher/welche/welches.”^4^ To take our diachronic approach into account we also included the relative particle “so” (Pfeifer, 1995) because it is more or less comparable to the English “that,” and has no additional meaning and is not bound to specific nouns.

Having established the kind of RC we want to investigate, we want to come to the main point: the position of RC. They can be placed adjacent and extraposed to their antecedent without changing the proposition of the sentence. The head noun can, for example, stand in the middle field while the RSB separates it from the RC (Birkner, 2008, p. 50; for the classification we use here, see section Method). Lehmann (1984) describes the extraposition as the process in which the RC is moved to the end of the sentence while Fritsch (1990, p. 114) specifies it as a movement to the right but no further than to the end of the smallest clause in which its antecedent is found. The most frequent explanations for the varying positioning are RC type, RC length, informational aspects, and the distance between RC and antecedent which is or could theoretically be covered.

We start with the RC type as it requires additional information. RC can be divided into restrictive and non-restrictive RC. Restrictive RC restricts the possible references of the antecedent (Birkner, 2008) when the antecedent is not sufficiently determined (3).


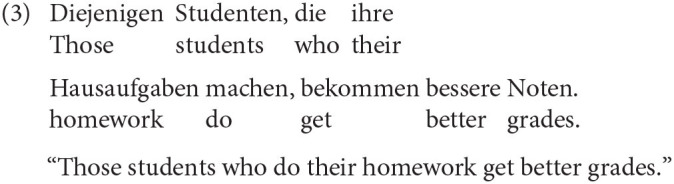


In this example, the RC limits the number of students getting good grades. Restrictive RC often follow determiners or pronouns like “jeder” (everybody) or “derjenige” [the one; Lehmann (1984); Fritsch (1990); Eisenberg (1999); Birkner (2008), among others]. There are also non-restrictive RC which only illustrate their antecedent and give further information about the antecedent. A German non-restrictive relative clause can be identified by adding “ja” or “eben” to the RC without creating a marked sentence. The antecedent of a non-restrictive RC is already completely determined (4)^5^.


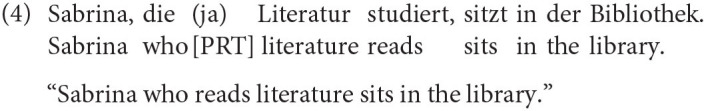


Restrictive RC is said to be more separable from their head nouns than non-restrictive ones. This can be attributed to the fact that the RC is necessary to complete the sentence. The recipient knows that the head noun is still incomplete and can therefore keep cognitive capacities free, which can be filled by the RC even at the end of the matrix clause. Non-restrictive RC may be too surprising when separated from the head noun over a long distance because they are not needed for the referential identification of the head noun. Though several scholars state but do not test, the influence of restrictiveness [Lehmann (1984); Fritsch (1990); Zifonun et al. (1997); Poschmann and Wagner (2016), among others], this could not be shown in the study of Poschmann and Wagner (2016) on RC where restrictiveness could not be determined as a predictor for extraposition.

The second, most frequent explanation for RC extraposition is length. On the basis of avoiding a stain of memory capacities and enabling effective processing of a sentence (Uszkoreit et al., 1998), RC length is often correlated with extraposition. Uszkoreit et al. (1998) found in their corpus study on German newspaper articles that extraposed RC in the PoF are on average one to three words longer than adjacent RC. The maximum distance is up to nine words but mostly varies between one and four words. In the latter case, the length of the RC becomes a more relevant factor. The longer an RC is, the more likely is its extraposition even over short distances. They showed that distance is the most crucial factor, followed by length (Uszkoreit et al., 1998, p. 130). Zifonun et al. (1997) also saw the length of extraposed material as one of the most important factors besides distance. One possible reason for this is combined with information disentanglement. Clauses are understood incrementally [Levy (2008), among others]. Words are ordered in a way that allows fast processing. On the one hand, this would mean that an RC should be adjacent as no dependencies must be kept in mind while processing the rest of the sentence (Hawkins, 1994; Gibson, 1998). On the other hand, Hawkins (1994) describes a complex interaction between the advantages and disadvantages of adjacency when the embedded material such as the RC is so complex that a recipient is no longer able to remember or incorporate the part of the clause which occurred in front of the RC. This holds true especially for RC placed in the middle field where the prefield and the first part of the middle field itself must be incorporated in order to understand the whole sentence. The longer the RC, the more cognitively challenging the processing of the RC and the matrix clause will be [see Gibson (1998) “memory load”].

The last reason frequently used to explain extraposition is information management. It is highly connected to the formerly mentioned memory load. Poschmann and Wagner (2016), for instance, showed a connection between the length of German RC and information structure. They saw a correlation between length and focus, as new material is usually longer than given material (Poschmann and Wagner, 2016, p. 1,022). They referred to the concept that easily accessible (that is: given or inferable) information is usually presented early in the sentence while new information tends to follow later.

Furthermore, the integration cost is influenced by the number of intervening new referents (Bader, 2014). They use a production experiment to find out how to focus, word order, and RC-type effect extraposition. In a second step, participants had to rate the acceptability of RC extraposition under the manipulation of word order, focus, and RC-Type. Though corpus data suggests an even distribution of RC, extraposition was rated worse than adjacency. This might be caused by long distances between antecedent and RC. RC with a wide focus is more acceptable than those with a narrow focus and the interaction between extraposition and focus shows that extraposition is rated better “when the NP it modifies or the RC itself is in focus” (Poschmann and Wagner, 2016, p. 1,057). They link their findings to other research investigating the influence of predictability, namely, the one presented by Levy et al. (2012) on English RC. The more constituents intervene between antecedent and RC, the more unlikely it becomes to find an extraposed RC. Levy et al. (2012, p. 29) show in their reading time experiments that “[r]elative clauses extraposed from simple [determiner + noun] NPs across a verb are harder to process than their corresponding *in situ* variants. RC extraposed from a direct object NP across a PP are harder to process than *in situ* RC modifying either the direct object (but following the PP) or the PP-internal NP. Nevertheless, a preceding context (specifically, NP-internal premodifiers) that sets up a strong expectation for a RC modifying a given noun can strongly facilitate comprehension of an extraposed RC modifying that noun.” They also assume that the scarcity of some collocations tested might also cause the shown difficulties of extraposed RC comprehension. We should keep in mind that one of their most precious findings is the influence of expectancy on reading times.

It is interesting to mention that the PoF and, with it, RC extraposition has undergone a changeover centuries. In Old High German, information structural considerations, namely, focus, are the most important next to restrictiveness which is also explained by information structure (Coniglio and Schlachter, 2015). The importance of information structure slowly decreases over time (Speyer, 2016) along with the usage of the PoF.

Even throughout ENHG itself, we see changes when the sentence brackets are finally established. According to Schildt (1976), from 1470 to 1530, 68% of the sentences in the corpus had no filled PoF, whereas from 1670 to 1730, this number decreased by 81%. Together with the decreasing frequency of PoF filling, this position also becomes more permissive in information structural terms. Early ENHG allowed especially new material in this position, while late ENHG does not make a real distinction between new and given material there. This might be a result of the not yet fully established sentence bracket structure in this period (Sahel, 2015, p. 168). The structure however becomes more pronounced over time (1650 to 1800) which is indicated by an increasing number of RC found there (Sahel, 2015, p. 172).

For the early New High German period, research, especially on RC extraposition, is rare. The sentence frame, finally established in the eighteenth century (Admoni, 1990; Konopka, 1996; Takada, 1998), can already be found in Old High German (OHG) but it was not as necessary as it is today and has begun to be in the eighteenth century. The material was more often placed on the borders of the clause and for various reasons (Paul, 2007). Besides the decline of phrasal material in the PoF, clauses were frequently placed there. Konopka (1996, p. 178) gives three reasons for placement of material in the postfield: “A. die Gestaltung der Informationsperspektive, B. die Sicherung der Textkonnexion, C. die Entlastung des überfüllten Satzrahmens.” (A. to shape the information perspective, B. to ensure text connectivity, C. to relieve strain on the sentence frame). Scholars from the seventeenth century agree with the latter: Though the sentence frame should be kept, the placement of clauses behind the RSB can ensure better processing of information [e.g., Schottelius (1641) in Takada (1998)].

In summary, bound RC can be placed adjacent or extraposed to their head noun. Reasons which have been proposed in the literature for the extraposition are the RC length, the distance to the head noun, restrictiveness, and information management. Closer examined, these reasons all refer to successful communication and information transmission.

### Information Density

Explaining, characterizing, and measuring successful communication and information transmission is the key feature of Information Density. A change in the position of certain linguistic material aims to improve communication by improving the transmission of information. In most literature on RC, information disentanglement or avoiding sentence fields that are too long are given as reasons for extraposition (cf. Section Relative Clauses). However, such approaches lack measurability, and even research results that deal with focus or givenness [Coniglio and Schlachter (2015) for example] can only include certain freedoms in the position in their considerations. Therefore, it is necessary to use a method that makes processing effort objectively calculable and does not differentiate between the information content of certain word forms. Such a theory is offered by Information Density theory of Shannon (1948).

In short, ID describes information as the probability of occurrence of a word in its context. The idea behind this is as follows: the less expectable a word is in its context, the more information it contains. Likelihood and information value correlate negatively with each other. The significance of this approach is that it offers explanatory potential for intra-linguistic variations, which, however, have no influence on the proposition of the sentence. According to Shannon (1948), the aim is not to write better messages, but to encode messages more effectively.

In almost all languages, there is a wide range of variations in the area of coding. In spoken language, the length of phones can be varied. In the field of morphology, speakers and writers can use abbreviations. Lexicology offers the possibility of variation between semantically very similar terms to express the same facts. For pragmatics, different reference expressions can be used to obtain variation in expressing the same facts. Syntactically, certain liberties in word order (see example 6) are offered (Gibson et al., 2019).

6) **Yesterday**, I gave him the book. → I gave him the book **yesterday**.

Both sender and recipient can select and decode different codes from a set of codes during the transmission of the message. All possible choices from this set of codes are equally probable according to Shannon (1948)^6^. The logarithmic function on the basis 2 is used as a mathematical description for the selection process (see below). Bits are thus the unit for information in context.

The signals and the coding must be adapted to the kind of transmission without exceeding the limits of the channel through which the message is sent and its specific capacity. The goal is to transfer the message into a language. This language already gives guidelines for the structure and thus, defines a natural frequency of certain elements. Both the sender and the receiver are aware of these structures. This leads either to time saving in the transmission of the message or to a less heavy load on the channel if the message sequence has been correctly encoded into the signal sequence (Shannon, 1948, p. 384). So, the transmission of the symbols is both incremental and dependent on the previous symbol and the symbol itself. The system of selecting the subsequent symbols can therefore be described as a stochastic process and is thus subject to the conditions of probability theory (Shannon, 1948). This can be represented as follows: p_i_(j), which describes the probability that j follows i (Shannon, 1948, p. 384).

If only the element itself is considered in its frequency, it is called unigram frequency. This is the simplest way to approach the stochastic process of element selection. However, this simple approach does not even come close to existing languages. For this purpose, more context must be considered, which then can be called bigram, trigram… n-gram. The larger the context, the more the results converge to the actual language, even if no attention is paid to conveying a specific content. “A sufficiently complex stochastic process will give a satisfactory representation of a discrete source” (Shannon, 1948, p. 386).

The core question that Shannon (1948) pursues consists of describing and mathematically explaining the conditions for optimal message transmission through a *noisy* channel. The considerations presented so far in the present work refer to a channel in which no interference is present, a so-called “noiseless channel” (Shannon, 1948, p. 19). However, this is only the case in a few situations. Nevertheless, most conversations are successful even if the speaker says something different than the receiver understands and the input is no longer equal to the output (Shannon, 1948, p. 19). This is highly dependent on context.

Certain words are more expectable in their context than other words. Let us consider (7):

7) You may now kiss the [bride].

This sentence might have been heard so often in wedding scenarios that the recipient has a strong expectation that the *bride* follows after a kiss and the definite article. So, the surprisal for the *bride* should be very low. Surprisal is usually calculated by the negative logarithm of the probability of an element given in a context: P(word) = –log_2_(*word*|*context*). Again, a distinction must be made as to how far the context is defined. In the case of unigram-surprise values, only the frequency of the element is relevant. In the case of bigram-surprise values, the probability of occurrence of the element before the considered element is included. Lastly, in the case of trigram-surprise values, the two preceding elements are included, etc.

Due to the very narrow context, even small changes can be decisive for other surprisal values. If the predicate was changed to *lecture* in example 9, the *bride* would no longer be a word marked with a low surprisal value as the *lecture* is more likely to occur in an educational context. These examples may be very simplified and may not capture the whole problem of positional variants. However, they do allow the first impression of ID theory and touch on a problem that can rightly be identified, namely, the strong focus of classical surprisal calculation on single words. Recent research shows that extralinguistic contexts such as script or world knowledge have an influence on the likelihood of a word and the difficulty in processing it [Ostermann (2020) among others]. However, it is precisely for historical contexts that the strong intra-linguistic orientation of theory of Shannon (1948) is useful since world knowledge can only be reconstructed to a limited extent and the knowledge of individual writers, on the other hand, can hardly be traced. The orientation toward purely written sources facilitates the objective evaluation of data.

Furthermore, the relationship between the predictability of linguistic material and efficient communication exists at all linguistic levels (Gibson et al., 2019), and a relationship between processing effort, i.e., psycholinguistic reality, and information density could be shown as well [Levy (2008) and others].

According to Levy (2008, p. 1,127), there is a probabilistic and expectation-based theory of syntactic understanding. Some syntactic structures consume more resources or memory than others. At the same time, human resources are limited which is why processing problems can occur in structures that consume a lot of resources. Therefore, the channel is virtually overloaded, so information is lost. Theories of syntactic processing gain importance. Thus, the understanding of information is based on different sources: structural, lexical, pragmatic, and discourse-based (Levy, 2008, p. 1,128). This results in a competition of similar analyses since these sources are combined for understanding (Jurafsky, 2003). The processing effort thus corresponds to the surprisal of a word. It is the interface between the linguistic representation during the comprehension of the sentence and the processing difficulties which can be found for a particular word within a sentence (Levy, 2008, p. 1,128). The recipient thereby preserves the complete set of the different, probable, and partially processed constituents from the already seen or heard input. They assign to it a possible probability distribution over the complete structure to which the already received constituents can expand. Surprisal is thus seen as the difficulty of replacing an old distribution with a new one (Levy, 2008, p. 1,132).

To facilitate communication, an even distribution of information is important at all linguistic levels, not only at the phoneme and grapheme but also at the syntactic level. Speakers design their utterances in such a way that there are no strong fluctuations in the information profile (Levy and Jaeger, 2007). This is achieved by exploiting the freedom of expression offered by languages or by omitting optional material. To prove this for the syntactic design of utterances, Levy and Jaeger (2007) investigated syntactic reductions and found them to be “a phenomenon in which speakers have the choice of either marking a phrase with an optional word, or leaving it unmarked” (Levy and Jaeger, 2007, p. 2). Their research topic is optional *that* in English RC. In their corpus study, they find that *that* is inserted when the surprisal on the first word of the RC would otherwise be too high, thereby exceeding the assumed channel capacity and causing a loss of information. Thus, they found the first evidence for what is known as the “Uniform Information Density Hypothesis.” It can be shown for both spoken and written English that speakers drop an optional relative pronoun, and this finding is also common across standard varieties (Jaeger, 2010, p. 163). This phenomenon and the UID can also be integrated into existing processing approaches and preferences. It can be compared both with “dependency processing accounts,” which assume that preference is given to variants that have shorter dependency relationships. They also take up the “Gesetz der wachsenden Glieder” (law of increasing constituents) by Behagel (1932). Furthermore, it concerns “alignment accounts” which regard access to referents as a major factor for linguistic preferences. These accounts rely on the conceptual accessibility and pre-mentioning of referents and can be combined with “availability accounts,” which focus more on the referent and claim that material that is cognitively available appears earlier in the sentence (Jaeger, 2010, p. 165). Incremental speech production is also related to this. What is available earlier can be expressed earlier, which in turn can be combined with the other approaches mentioned above.

While the language processing system works basically incrementally, at least for the hearer, there is still the need to keep the elements of a clause together in the working memory as syntactic dependencies must be reconstructed by the hearer and the verb valency has to be checked. Therefore, another factor in the calculation must be the sum of the surprisal values of the individual lexical items within a clause as they must be related to each other and thus, to some degree, processed together. It is reasonable to assume that a clause containing some words with high surprisal is a whole lot more difficult to process than a clause containing only words with low or medium surprisal values. To account for this fact, we use two measures that are derived from surprisal: the *cumulative surprisal* of a clause is the sum of all individual surprisal values of the words in the clause, and the *mean surprisal* is the arithmetic mean of the surprisal values in a clause, that is, cumulative surprisal divided by the number of words in the clause (cf. Section Methodological Considerations About RC Extraposition and ID).

In summary, ID according to Shannon (1948) determines the information content of a word in a certain context and links this information content to the likelihood of the word in the context. The surprisal value is calculated by the logarithmic function and expressed in bits. The aim of ID theory is to provide a descriptor for the optimal encoding of a message and thus, to be able to demonstrably describe how information loss can be prevented. In the classical method of calculation with n-grams, *all* words, namely, content and function words, are considered in the calculation of the surprisal values, whereas in classical information-structural studies often only content words are considered. Thus, no positional changes can already lead to visible effects. A description of why and how this concept is applied to RC follows in the methodology section.

## Corpus and Method

This section presents the basis for our research. We will present the corpus we used and provide further reasons for our decision to work on early New High German. The second part of the section is concerned with our method. We present our annotation process and our language model. The section is closed by an explanation of the predictors we consider relevant for extraposition. A special goal is to show that while length might have already proven important for extraposition, it is not necessarily the best predictor for extraposition. Using ID as a predictor instead might lead to a different conclusion. We are aware of the rather exploratory character of the study.

### Corpus

Our corpus is built on texts from the *Deutsches Textarchiv* (DTA). The DTA is a collection of texts from different genres and periods ranging from the seventeenth to the twentieth century. Balanced samples from newspapers, novels, literature for a specific purpose (“Gebrauchsliteratur”), and scientific texts provide an overview of the German language development. A major advantage of the DTA is the preprocessing of the texts. They are tokenized, normalized, lemmatized, and POS-tagged albeit in a rather poor quality which complicates and even prevents automatic annotation.^7^

The DTA is the only database with such a high variety of genres that includes scientific, namely medical texts. Before the seventeenth century, German scientists used to publish their findings in Latin so that a German tradition of scientific writing in the native language of an individual developed only at the end of the ENHG period. Even then, the publishing process did not resemble the one we know today but consisted of letters to interested colleges. This puts this genre in the field of tension between different registers, namely written and oral discourse modes (Koch and Oesterreicher, 2007). Despite being a written form of communication, letters tend to be closer to the oral discourse mode than the written discourse mode. Typical examples are addressing the addressee or, according to the theory, placing more material in the PoF. At the same time, these authors might be influenced by the former Latin tradition with elaborate rules on how to write prose and might be influenced by that. Because (written) Latin does not have a sentence frame like German and has widespread dependencies that would strain the parsing capacities of a German native speaker, this might contradict the optimal distribution of information when a clause is written in a more Latin-like style at the beginning of seventeenth century. This strain between the letter style and the former Latin tradition might result in longer, intertwined clauses that decrease over the centuries. In the nineteenth century, however, texts might also resemble a more modern scientific style with shorter, less intertwined clauses. Therefore, it is also important to have a data basis that spreads over the centuries like the one provided by the DTA.

As we are not interested in grammatical but in lexical predictability,^8^ lemmatization is a crucial factor for our analysis. Due to the non-standardized orthography in ENHG, normalization is an important step. The Language Model (see Section Language Model) would not capture the same word when it is spelled in different ways. Because words appear in different inflected forms, however, normalized data is not sufficient for the language models either, but we need lemmatized data to capture all instances of a given word in whatever form they appear and in whatever way they are written.

Our corpus from the DTA used in this study consists of the nine medical texts from 1650 to 1900 with 841,877 tokens^9^. The texts were chosen arbitrarily while translated texts were excluded. The 250-year time span was divided into 50-year-steps to account for possible changes in language use, orthography, and writing style preferences which are highly relevant for the calculation of the language model (section Language Model). The corpus under study consists of the following texts (Table 1).

**Table 1:** Corpus.

**Period**	**References**
1650–1700	Purmann, 1680; Abel, 1699
1700–1750	Unzer, 1746
1750–1800	Gall, 1791
1800–1850	Reil, 1803; Carus, 1820
1850–1900	Ludwig, 1852; Koch, 1878; Kraepelin, 1892

### Methods

#### Annotation

We want to emphasize that all annotations were made manually due to the poor POS-tagging of the DTA. We used WebAnno (Eckart de Castilho et al., 2016) for the annotation^10^.

We manually annotated the following features in the corpus: the RC,^11^ their position as described below, their antecedent, that is, the noun or pronoun the RC depends on, and their type (restrictive vs. non-restrictive). To annotate the RC type, we determined whether the RC is necessary to clearly identify its antecedent. The main criterion to determine the restrictiveness of the RC is whether the antecedent can be completely and uniquely identified without the RC. Certain hints at restrictiveness are, for example, given by certain determiners (e.g., *derjenige*, “the one”). In the case of non-restrictive relative clauses, we are confronted with the problem that we cannot be sure whether the insertion of “ja/eben” would have been marked for ENHG writers. Because of the language period, the annotation of restrictiveness was not possible in every case because we cannot reproduce the world knowledge of, for example, a seventeenth century writer. In these cases, the type was not annotated (NA). Furthermore, we annotated the Left and Right Sentence Brackets (LSB and RSB) following Wöllstein (2014). The categorization of the sentence brackets is necessary to determine whether an RC is extraposed or not. The length of the RC and the distance between antecedent and the first word of the RC were both calculated automatically and not manually annotated.

We only annotated RC and not whole sentences. We are aware that we should also look at the ID profile of the whole sentence, but again the DTA provides some disadvantages. Due to the rather irrelevant punctuation and the practice in ENHG to sometimes end a sentence with a semicolon, so not even a human reader can be sure whether that really marks the end of a sentence or just a clause, the automatic sentence recognition fails. As a result, some sentences are incomplete and WebAnno does not allow our annotation to continue over sentence boundaries, whereas others include several sentences and are marked as one. As we have not yet annotated the sentence boundaries manually, only the RC, themselves, are considered for the results. The number of RC we found in the corpus is given in Table 2 in the following section.

**Table 2:** Language model.

**Period**	**Training data (in token)**	**Test data (in token)**	**OOV-ratio**	**Number of RC (extraposed RC)**
1650–1700	2,107,590	48,1693	8.93%	240 (116, 48%)
1700–1750	1,481,259	39,251	6%	680 (363, 53%)
1750–1800	2,572,263	26,325	14.72%	375 (130, 35%)
1800–1850	998,639	16,757	6.28%	1,023 (573, 56%)
1850–1900	1,270,561	29,060	12.13%	925 (467, 50%)

The most relevant factor is, as mentioned before, the adjacency of RC and head nouns. When both are in the prefield or in the middle field framed by both sentence brackets they are clearly determined as embedded or *in situ* (8a). Also, when the RC (underlined) is behind the RSB and the head noun (bold) is either in the prefield or, more often, in the middle field, it is without a doubt an extraposed RC (8b). But there are also cases in which the determination of the RC position is not as easy. The RSB can remain empty but still build the end of the clause. Two special cases arise when the RC is at the end of the clause and adjacent to its head noun (8c), we called the RC ambiguous and excluded it from the analysis because we cannot rule out that there has not been a movement over the empty RSB. But when there is a material other than the RSB intervening between the head noun and RC we classified the RC as extraposed. While we can, strictly speaking, not be sure whether the RC is actually in the PoF (8d), the fact that the RC is no longer adjacent to the head is crucial and outweighs the uncertainty.


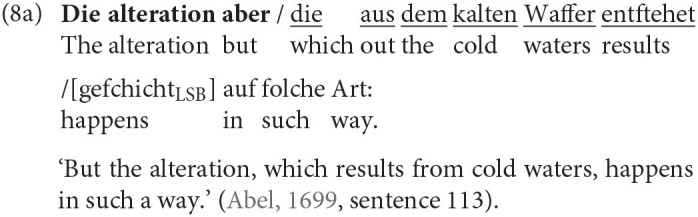



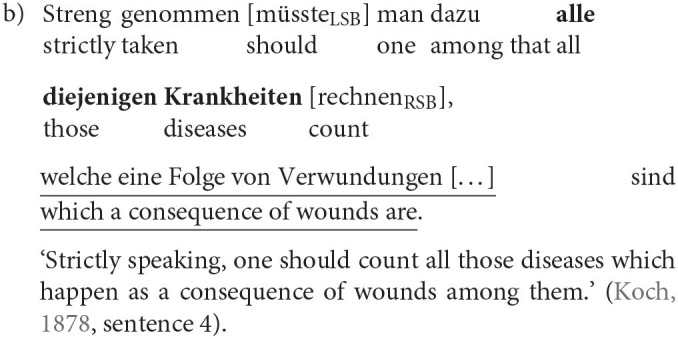



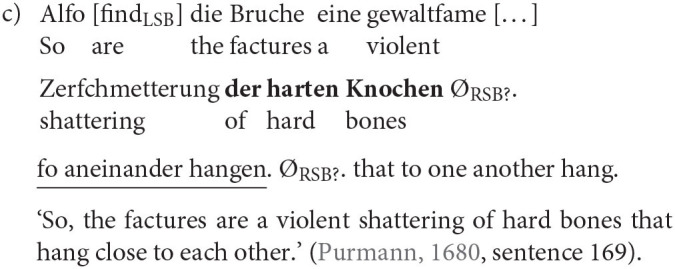



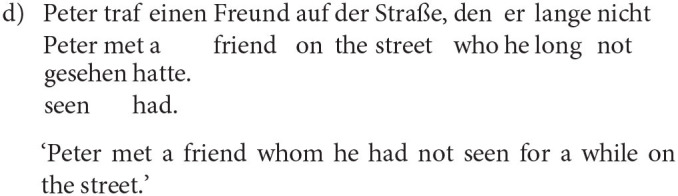


The software R (R Core Team, 2018) was used for further data processing. All sentences not including RC were excluded and punctuation marks were removed because rules for the placement of punctuation marks had not yet been established, and they were often placed according to personal preferences of the authors so that an additional meaning or advantage of their inclusion could not be found. Then, we calculated the skip-gram language model on every remaining word and checked for the influence of RC length, type, cumulative, and mean surprisal. Note that RC length was calculated automatically with R. We will provide more details regarding our motivation for the analysis in the following sections. Since the data is not very balanced, we perform the statistical analysis not only on the whole data which would not be feasible for the second hypothesis anyway but on every 50-year timespan separately (see Section RC per Period).

#### Language Model

In the next step, we calculated a Language Model (Hale, 2001) and a skip-gram Language Model with a 2-skip-bigram (Guthrie et al., 2006) for every 50 years on the lemma layer of the corpus using an SFB-intern tool. Skip-grams were chosen over bigrams because they do not only take immediately adjacent words for the model but allow tokens to be skipped to create trigrams, thus capturing the context better and achieving better coverage of the data. This is especially useful when the training data varies from the test data and increasing coverage of n-grams cannot be assumed (Guthrie et al., 2006, p. 1,223). The model was trained on those scientific texts in the DTA that were not included in the test data.^12^

Training data is used to gain estimated values over the following words given its context using a hidden Markov model. It states that the probability of a future unit can be predicted without looking too far into history (Mürmann, 2014). For languages, this means that not every linguistic utterance ever produced must be included in the calculation, but that a part of the linguistic utterances is sufficient to be able to make acceptable statements. The surprisal value of a word is obtained by calculating the probabilities of its occurrence and mapping them to the test data. This is done using the Maximum Likelihood Estimate: “the maximum likelihood estimate is so called because it is the choice of parameter values which gives the highest probability to the training corpus. [.] It does not waste any probability mass on events that are not in the training corpus, but rather it makes the probability of observed events as high as it can subject to the normal stochastic constraints.” (Manning and Schütze, 1999, p. 198). Further smoothing methods are applied to enable the model to give an estimate to tokens unseen in the training data but are used in the test data.

The Language Models were calculated without punctuation marks since they are not meant for ENHG (see above). The following Table 2 sums up the corpus including training data, out-of-vocabulary-token-ratio, and the number of RC.

#### Methodological Considerations About RC Extraposition and ID

The length of the RC is one of the most frequent factors used to explain extraposition. Various studies prove this for both German (e.g., Uszkoreit et al., 1998; Poschmann and Wagner, 2016) and English (e.g., Levy et al., 2012). At the same time, however, the factor of informativeness of the RC is also repeatedly used as an approach in theoretical and experimental studies. Intuitively, the two concepts do not contradict each other. The more words are available in a sentence, the more information it can contain. The more information there is, the more cognitive capacities are needed to process the sentence. However, if, at the same time, cognitive capacities are also used on other processing issues, such as the comprehension of a complex middle field of the matrix sentence, an RC occurring there could cause an overload of the available cognitive capacities. In this case, communication should fail. This approach is represented by the well-known theories on the extraposition of RC presented by Hawkins (1994) and Gibson (1998). Both, as mentioned above, limit themselves to measuring complexity by the number of words.

However, the information density approach of Shannon (1948) and more recent research by Levy and Jaeger (2007); Levy (2008); Jaeger (2010), and others show that an increase in length, i.e., the addition of words, does not necessarily equate to a significant increase in information if the information is understood as the predictability of a word in context. Both the immediate context of a word and the extended context can reduce the probability of occurrence of a word. This, in turn, would reduce the information content of the specific word and could eventually lead to a reduction in the overall information content of the sentence despite a higher number of words. A simple example (9) illustrates this:


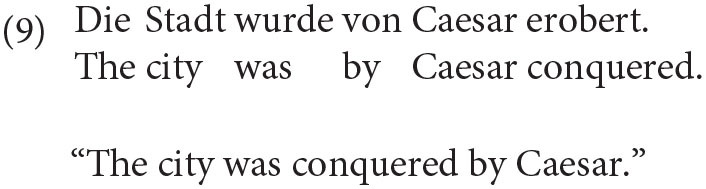


This sentence contains five words. Without a larger context, the information content of Caesar should be quite high. If you now add words at various points and thus increase the length of the sentence, you simultaneously reduce the informativeness of various words.


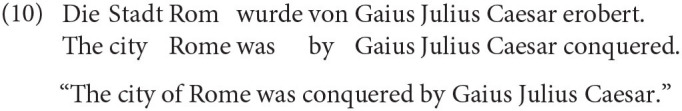


The sentence (10) is extended to nine words. At the same time, both the mention of Rome and the mention of first and gentil names of Caesar should ensure that the likelihood of “Caesar” increases enormously with the preceding “Gaius Julius” and that the negatively correlated surprisal value falls. Theoretically, but more difficult to prove, depending on the language model used, even the mention of Rome can cause the full name to be assigned lower surprisal values since Caesar and Rome are closely connected. The added words can therefore ensure that the sentence is easier to process through the selective reduction of the information content, although it has become longer at the same time.

This effect was demonstrated by Levy and Jaeger (2007), among others, and, subsequently, many times by Jaeger (2010) when investigating optional elements in a sentence, namely, the optional use of *that* as an RC introducer. In less expectable contexts, the use of the relative pronoun can ensure that an overwhelmingly high processing load on the first word of the RC is reduced. It can therefore be stated at this point that informativeness and length do not necessarily have to be positively correlated with each other and that a separate consideration of the two is appropriate. These theoretical considerations lead to two possibilities for calculating the information density: We use cumulative and mean surprisal values.

The justification for the cumulative surprisal value lies in the parallel processing of information (e.g., McClelland and Elman, 1986). The entire information density theory of Shannon (1948) is based on the incremental approach. Words are processed one after the other and the likelihood of a word results from its context. Previous theories and experimental methods that measure processing difficulties mostly work with local phenomena. Bigram language models and, to a certain extent, skipgrams are strongly dependent on a narrowly defined context. Reading time studies measure delays on specific individual words and focus, simply put, on problems at individual points. These methods are not well-suited to determine the total processing effort of a sentence. Because other factors are also relevant for understanding such as parallel processing of grammatical structures or the inclusion of different sources (e.g., Cutler, 2008), it is important to find a model that approaches the total processing effort but is also usable for corpus data. The sum of all surprisal values in a clause, or even just a construction, can be understood as an approximation. The idea behind this is the following: the cognitive capacities are neither immediately free after processing a word nor are they immediately available again. Instead, they form a kind of pedestal that grows larger with each additional word depending on its surprisal. Only when the construction is completed does the full processing capacity become free again and the filling process of the pedestal can begin again at a low level.

However, the calculation of the sum leads to some problems. Even though it was argued above that more words do not automatically have to lead to more information on certain words and thus perhaps also in the total set, it can be assumed that the addition of surprisal values correlates with the length of the material studied. The more values are added, the larger the cumulative surprisal value can become. This would only not be the case if surprisal values are zero or negative, which would require perfect redundancy. However, this is not the case in languages (Shannon, 1948), which is why it is impossible to achieve a reduction in the cumulative surprisal value with an increase in length.

To reduce the influence of the length on the processing effort, the mean surprisal value must be calculated. The justification results from the calculation of the arithmetic mean value. A correlation between length and mean surprisal should no longer be found, length is practically factored out. Because the mean value is strongly influenced by outliers, an RC consisting of a few very surprising words could have a high average surprisal value which, according to our theory, should produce a higher processing effort and favor extraposition.

To illustrate, example (11a) shows an extraposed RC from 1,680 with a cumulative surprisal value of 24.13 but only six words, whereas (11b) shows an embedded RC from 1820 with 14 words and a cumulative surprisal value of 42.71, which is within the first quantile of cumulative surprisal values for embedded RC with more than 12 words.


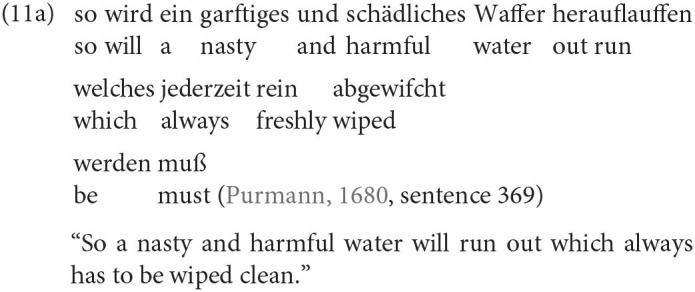



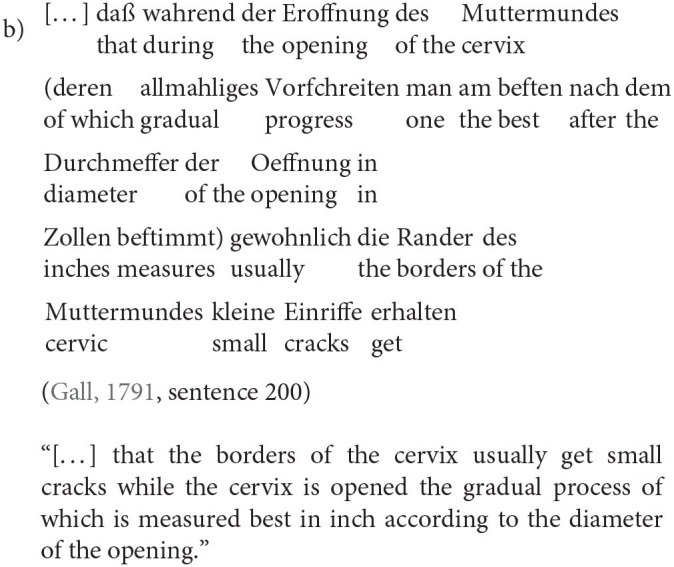


Neither mean nor cumulative surprisal measurements have been previously used to explain RC extraposition. Both methods are somewhat interrelated and cannot be evaluated as better or worse suited to describe the processing effort for a construction purely based on preliminary theoretical considerations. Both involve the complete set of surprisal values, rather than focusing only on a local phenomenon and the increase or decrease of the likelihood of a word at that point. To find evidence for the previously postulated distinction between ID and length, a first section will evaluate some descriptive statistics before using linear regression (glm, R Core Team, 2018, Base-Package) to determine the best predictors.

## Results: Information Density and Length

### Whole Data

**Figure 1:**
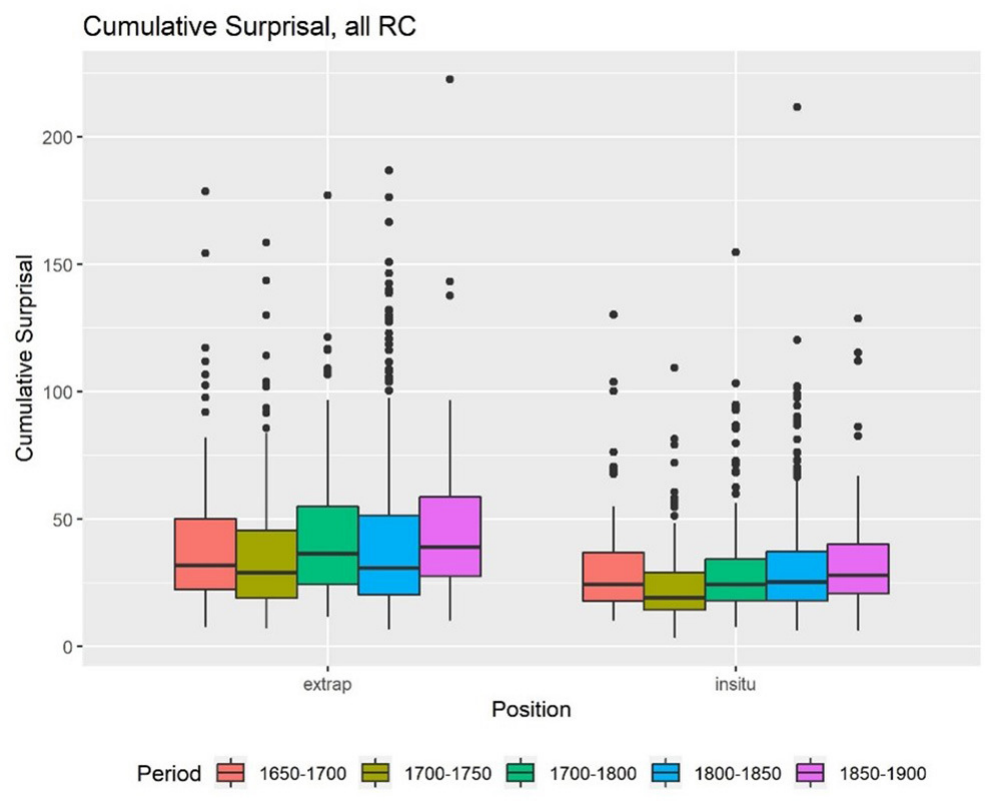
Cumulative surprisal.

**Figure 2:**
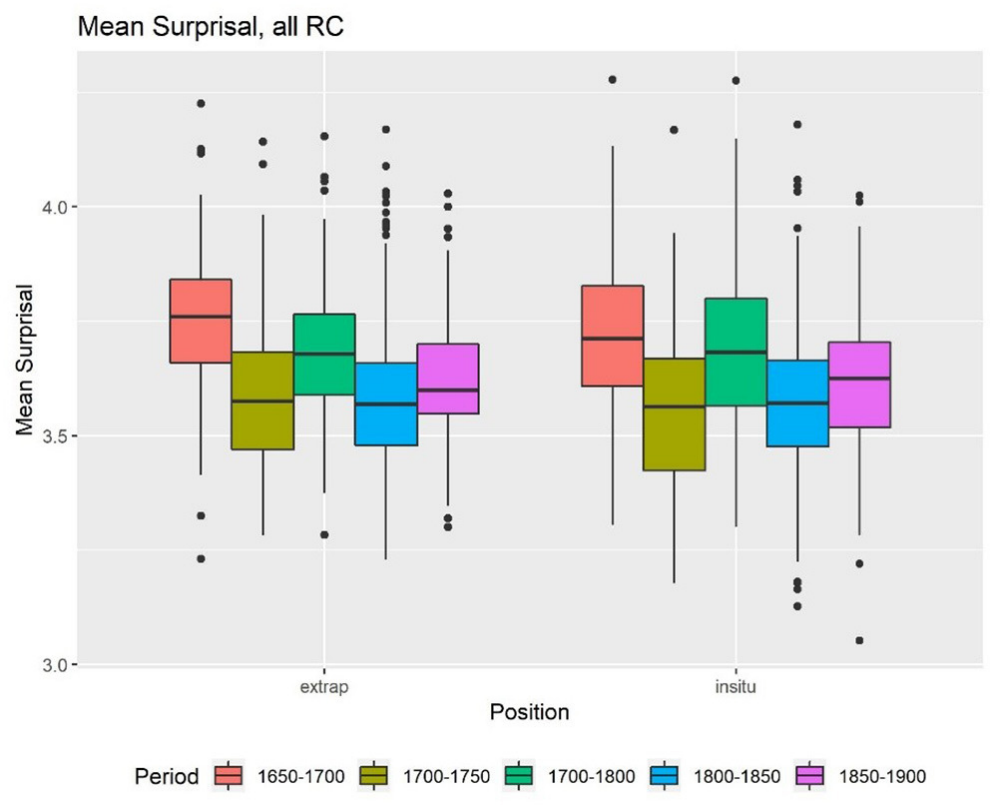
Mean surprisal.

The first factor, which is also most relevant for the hypothesis, is ID. First, we calculated the accumulated, mean Skip-gram surprisal values, and the length for all time periods. The descriptive statistics show that there is in fact a difference between the cumulative surprisal values of extraposed and embedded RC (Figure 1). In general, extraposed RC seem to have a higher cumulative surprisal value than embedded RC, which are labeled “*in situ*” in all graphs. The mean surprisal values for the RC in both positions do not appear to differ that much (Figure 2). In both cases, we find a lot of outliers but little differences within the centuries.

Problems arise when we check for the influence of length and the assumed correlation between length and cumulative surprisal values. The correlation value between length and cumulative surprisal values is 0.98, which suggests a very strong correlation. The longer an RC is, the higher are its surprisal values. But for the mean surprisal values, we do not find this correlation (*r* = 0.00052). Thus, there is no correlation between the length of an RC and its mean surprisal value, and only an insignificant correlation between the two surprisal values (*r* = 0.1283636).

Checking the predictors using logistic regression (R Core Team, 2018)^13^ and if writing styles (that is authors) do not influence extraposition, we only find an expected (cf. Section Methodological Considerations About RC Extraposition and ID) and slightly significant interaction between length and cumulative surprisal (*z* = 2.571, *p* < 0.05). All other predictors are not significant. In a second step, we removed the correlation between type and length, which does not change any of the parameters and does not lead to a better model. Next, the interaction between mean surprisal and length was removed, which presents the cumulative surprisal value as significant (*z* = −2.417, *p* < 0.05). The rational likelihood analysis conducted using ANOVA (R Core Team, 2018) shows that the model transformation is permissible. In the next step, we removed the interaction between cumulative surprisal and type, resulting in an interaction between cumulative and mean surprisal (*z* = 1.982, *p* < 0.5). The last interaction between type and mean surprisal was then cut along with the interaction between cumulative and mean surprisal value. The rational likelihood analysis granted this procedure as well. Our final model (Table 3) consists of the predictors cumulative surprisal (*z* = −2.23, *p* < 0.05), mean surprisal (*z* = 1.511, *p* = 0.13), length (*z* = 0.56, *p* = 0.57), type (*z* = 1.74, *p* < 0.1), and the interaction between cumulative surprisal and length (*z* = 2.8, *p* < 0.001). A further reduction of the model does not lead to a significantly better model. The interaction can be explained by the close connection between the calculation method and length. That makes it more difficult to determine whether length or surprisal is more influential. This result is interesting for several reasons. First, it shows a correlation between cumulative surprisal values and extraposition in a way we expected. But the influence of RC type, that is, restrictiveness contradicts previous statements in the literature. Restrictive RC are more likely to be embedded in our data whereas former research proposes the opposite.

**Table 3:** Most influential effects in the final linear regression model (GLM) predicting position from surprisal values.

**Predictor**	***z*-value**	***p*-value**
Cumulative surprisal	−2.23	<0.05*
Type	1.74	<0.1
Cumulative surprisal: length	2.8	<0.001**

The removal of the interaction, though not covered by the rational likelihood analysis, lowers the *p*-values and marks length (*p* = 0.38) and mean surprisal (*p* = 0.14) as non-influential. If we drop length as well, cumulative surprisal seems to be the best predictor for extraposition (*z* = −9.543, *p* < 0.001), only followed by the RC type (*z* = 1.74, *p* < 0.1). We can therefore say that cumulative surprisal does seem to be highly correlated with extraposition, and we can therefore conclude that ID seems to be a better predictor than length. Still, one must be careful in making assumptions because this puts a time span of more than 200 years under consideration and the interaction in the model, which explains our data best, should not be forgotten. Therefore, the following sections will concentrate on the results for the 50-year timespan which were already used to calculate the Language Model. We can thus prevent the results from being skewed because of slightly imbalanced data.

### RC per Period

Having established that ID seems to, indeed, have an influence on extraposition, the next step is to check whether this influence changes over the course of 250 years. Therefore, the corpus was split into five parts, each representing a 50-year timespan (Table 4).

**Table 4:** Descriptive statistics.

**Period**	**Number of RC (extraposed RC)**	**Min./Max. cumulative surprisal (mean)**	**Min./Max. mean surprisal (mean)**	**Min. /Max. length (mean)**	**Min./Max. distance (mean)**
1650–1700	240 (116, 48%)	6.697/242.55 (42.066)	3.19/4.36 (3.74)	3/58 (10.42)	1/10 (2.38)
1700–1750	680 (363, 53%)	6.7/177.55 (34.99)	3.19/4.41 (3.67)	2/50 (9.7)	1/17 (2.08)
1750–1800	375(130, 35%)	7.17/216.88 (41.45)	3.291/4.260 (3.716)	3/37 (11.14)	1/7 (1.87)
1800–1850	1023 (573, 56%)	6.36/211.69 (39.39)	3.13/4.18 (3.57)	2/58 (10.42)	1/14 (1.78)
1850–1900	925 (467, 50%)	6.87/222.628 (40.10)	2.91/4.09 (3.62)	3/66 (11.58)	1/11 (1.74)

In the first timespan (*1650–1700*), 240 RC were found, 116 (48%) of them, are extraposed. The cumulative surprisal values range from 6.697 to 242.55 with a mean of 42.07. Length is closely related to the cumulative surprisal value. The RC length differs between 3 and 58 words with a mean of 10.42. In the cases of very long RC with high cumulative surprisal values, the RC is not only complex in words but also in its grammatical complexity. The RC contains other subordinate clauses which are so closely linked to the content of the RC in question that it would be wrong to disregard the dependent subordinate clauses because this would not capture the whole message and its specific coding. This procedure was used for all other periods as well (12).


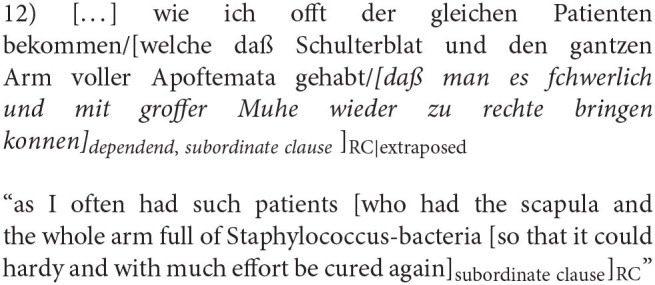


As expected, the mean surprisal values do not have such a great variation. They only vary between 3.19 and 4.36 with a mean of 3.74. The distance between an extraposed RC and its head noun fluctuates between 1 and 10 with a mean of 2.38. It is interesting to notice that the material over which the RC is extraposed is mainly built by the RSB, one single constituent or one constituent, and the RSB. In the cases of a distance >4 words, we can still say that the RC is only moved over one constituent though this constituent contains a whole clause. Even when the head noun wsas in the prefield, only the sentence brackets and one other constituent interfered between it and the RC. In other cases, the large distance was caused by references when findings of other scientists were quoted. The distance was only calculated for extraposed RC. Thus, it is only included in the descriptive statistics because we are yet unable to reliably calculate the hypothetical distance over which embedded RC could be moved to land at the end of a clause due to the poor processing of DTA data and the uncertainty of clause boundaries as described in Section Annotation.

For the time span from 1700 to 1750, we find 680 RC in total, and 363 (53%) of them are extraposed ones. With 6.7, their smallest cumulative surprisal is slightly higher than the one from the 1650's period while the largest cumulative surprisal is only 177.55 bits. Its mean is 34.99 bits. The closely related length varies between 2 and 50 with a mean of 9.7. The mean surprisal values differ from 3.19 to 4.41 with a mean of 3.67, and the distance varies between 1 and 17 with a mean of 2.08. Again, the large value of this variable is caused by interfering sentences such as parentheses. It becomes clear that the difference between the 1650's and 1700's RC is rather small. We find more RC, but their values mostly differ in the maximum cumulative surprisal value which might indicate a higher amount of information in RC in the late seventeenth century.

This changes again in the period of 1750 to 1800. We find slightly less RC with 375 and only 130 extraposed RC. That is the smallest percentage of RC in the whole corpus (35%). The smallest cumulative surprisal value is 7.17, the largest is 216.88, and the mean is 41.45. RC seems to be able to convey more information, compared to the previous period though not as much as in the first period. This is highly interesting because, at the same time, the range of length of RC decreases noticeably. Particularly, even the shortest RC contains six words while the longest on the other hand contains 13 words. The inner complexity of the RC decreases apparently in this period. The distance between the head noun and RC is smaller than in other periods as well. It ranges from 1 to 7 with a mean of 1.87. Once more, the mean surprisal values do not have a big variability. The smallest mean is 3.29, the biggest is 4.26, and the mean is 3.72.

The last two periods contain the highest number of RC. In the 1800 to 1850 period, 1,023 RCs were detected, among them 56% extraposed RC (573). The cumulative surprisal values range from 6.36 to 211.69 bits with a mean of 39.39. The length resembles the length of the early periods with a variety between 2 and 58, and an average of 10.42. The same holds for the distance between antecedent and RC. It varies again between 1 and 14. The longest distances are produced by interfering parentheses, clauses, and by references which were not excluded. The mean surprisal is rather constant again, ranging from 3.13 to 4.18.

The last period (1850 to 1900) contains 925 RC and 467 extraposed RC which corresponds to 50%. We find the second highest maximum cumulative surprisal values in this period (222.68) and the third highest minimal cumulative surprisal value (6.87). The average cumulative surprisal is 40.10. Another peak value is reached in the RC length, which ranges from 3 to 66 and achieves a mean of 11.58. The outlier RC of over 60 words is once more very complex and contains several dependent subordinate clauses. This period does not show any more extraordinary values in the distance which covers a span from 1 to 11 and is 1.74 words long on average. The mean surprisal values vary between 2.91 and 4.09. The minimum mean surprisal value is the smallest in our corpus (2.91).

Having collected the data, the next step is to check which factor influences the RC position to which amount. The procedure for the regression analysis of the different timespans follows the procedure presented for the whole data. We included cumulative, mean surprisal, and the length of the material into a linear regression model (glm, R Core Team, 2018, Base Package)^14^ and then conducted a backward model procedure using ANOVA (R Core Team, 2018). Restrictiveness was excluded since it was only marginally influential in the analysis of the whole data and could not be determined in many cases. Further explanations for the removal will be presented in section Discussion.

For the period **1650 to 1700**, the first model which includes all parameters and interactions does not show any significant predictors. This does not change until we remove all interactions and the mean surprisal values. Thus, the cumulative surprisal value is marginally significant (*z* = −1.8, *p* < 0.1) and claims that RC with higher cumulative surprisal values are more likely to be extraposed, whereas length is not only not significant but presents us with a value contradicting the idea that longer RC are placed in the post field (Table 5). Our data suggests the opposite. The first period, therefore, provides evidence for our first hypothesis.

**Table 5:** Most influential effects in the final GLM predicting position, 1650–1700.

**Predictor**	***z*-value**	***p*-value**
Cumulative surprisal	−2.669	<0.01**
Length	2.268	<0.05*

The **period of 1700 to 1750** presents a slightly significant value for the mean surprisal values (*z* = −1.71, *p* < 0.1) in the model with all predictors. The backward model selection allows us to exclude the interactions between cumulative and mean surprisal, and the one between cumulative surprisal and length. The result improves the significance of the mean surprisal (*z* = −2.076, *p* < 0.05) and adds a slightly significant interaction between length and mean surprisal (*z* = 1.718, *p* < 0.1). Longer RC has higher surprisal values, but this interaction is only marginal. To remove this interaction from the model is possible, but the results will have insignificant values. Therefore, the interaction between mean surprisal and length is included in the model again, but the cumulative surprisal must be excluded.

The resulting model succeeds better in explaining the results. Having a model consisting of mean surprisal, length, and their interaction presents the following results: RC with a high mean surprisal value is more likely to be extraposed (*z* = −2.147, *p* < 0.05) and length gains in influence (*z* = −1.686, *p* < 0.1). The interaction shows a *p*-value over 0.1 now (*z* = 1.541, *p* = 0.1234). That is why the interaction is no longer included in the model. Our final model incorporates length and mean surprisal and is significantly better than a model without length (*p* < 0.001). Though mean surprisal values are still marginally influential (*z* = −1.693, *p* < 0.1), length is the best predictor for extraposition (*z* = −3.961, *p* < 0.001) in this case. This result stands in contrast to our finding for the first period and to our first hypothesis (Table 6). Further considerations on this period will be presented in Section Discussion.

**Table 6:** Most influential effects in the final GLM predicting Position, 1700–1750.

**Predictor**	***z*-value**	***p*-value**
Mean surprisal	−1.693	<0.1
Length	−3.961	<0.01**

The picture differs in the **period of 1750 to 1800**. As in the period of 1650 to 1700, the first model which incorporates all variables and interactions has no significant predictors. Models with interactions do not explain the phenomenon of extraposition sufficiently, and even the model with only length, cumulative and mean surprisal does not achieve this. We removed length as well in order to find a model which is able to explain the phenomenon. The result is highly significant for cumulative surprisal values. The higher the surprisal value the more likely the RC is to be extraposed (*z* = −4.471, *p* < 0.001). Mean surprisal values do not show this correlation (*z* = 0.186, *p* = 0.052). The backward model procedure shows that a model with mean surprisal does not explain the data significantly better (*p* = 0.8079). So, in this period, we find only a significant correlation between cumulative surprisal and extraposition and therefore evidence for the first hypothesis (Table 7).

**Table 7:** Most influential effects in the final GLM predicting position, 1750–1800.

**Predictor**	***z*-value**	***p*-value**
Cumulative surprisal	−4.471	<0.01**
Mean surprisal	−0.186	<0.1

For the next **period of 1800 to 1850** similar findings can be presented. No variable in the model produces significant results when it is put in a model with all interactions or when the model includes all variables. As in the data from 1750 to 1800, we do not find significant results by incorporating cumulative and mean surprisal and length. In this model, length presents the highest *p*-value (*z* = −0.096, *p* = 0.923). Neither cumulative (*z* = −0.397, *p* = 0.691) nor mean surprisal (*z* = 0.68, *p* = 0.492) seem to be influential. We, therefore, exclude length and gain a model which presents a highly significant correlation (*z* = −5.474, *p* < 0.001) for the cumulative surprisal values and no correlation for mean surprisal (*z* = 0.853, *p* = 0.394). This slightly more complex model does not explain the data better than a model only including cumulative surprisal values. Again, we find evidence for our hypothesis: high cumulative surprisal values favor extraposition (Table 8).

**Table 8:** Most influential effects in the final GLM predicting position, 1800–1850.

**Predictor**	***z*-value**	***p*-value**
Cumulative surprisal	−5.474	<0.001***
Mean surprisal	0.853	=0.394

The last **period (1850 to 1900)** is the first to present a significant interaction in the model with all variables and interactions. This interaction happens between cumulative surprisal values and length (2.057, *p* < 0.05). No other significant correlations or interactions are found. We, therefore, remove the interaction between mean surprisal and length. This reduces the interaction between cumulative surprisal and length to a slightly significant one (*z* =1.865, *p* < 0.1) and introduces a slightly significant cumulative surprisal value (*z* = −1.768, *p* < 0.1) as well. The following removal of the interaction between mean and cumulative surprisal values shows the influence of cumulative (*z* = −1.8, *p* < 0.1), mean surprisal (*z* = 1.736, *p* < 0.1), and a highly significant interaction between cumulative surprisal and length (*z* = 2.67, *p* < 0.05) (Table 9). A further reduction of the model does not lead to a model which explains the data any better. If we still take that step and exclude length, the only predictor in the model which does not show a significant correlation, the model results resemble those from other periods (Table 10) wherein cumulative surprisal is highly significant (*z* = −8.027, *p* < 0.001) and mean surprisal value marginally significant (*z* = 1.835, *p* < 0.1). But we must keep in mind that this model is not a significantly better model than the one including length and its interaction with cumulative surprisal values. In the last period, the influence of surprisal on extraposition seems to be only marginal but still stronger than the influence of length. But its strong interaction with cumulative surprisal might also influence these results.

**Table 9:** Most influential effects in the final GLM predicting position, 1850–1900.

**Predictor**	***z*-value**	***p*-value**
Cumulative surprisal	−1.8	<0.1
Mean surprisal	−1.736	<0.1
Cumulatvie surprisal: length	2.67	<0.05**

**Table 10:** Most influential effects in the final GLM predicting position, after removing interactions, 1850–1900.

**Predictor**	***z*-value**	***p*-value**
Cumulative surprisal	−8.027	<0.001***
Mean surprisal	−1.835	<0.1

We want to sum up our findings: For all periods except for the timespan 1700 to 1750, we find an influence of ID which exceeds the influence of length. For the timespan 1850 to 1900, our corpus does not allow a distinction between length and ID. Therefore, we must be careful with the data interpretation though removing length results in significant data for ID. All other periods provide evidence for our first hypothesis in which RC with higher cumulative surprisal values is more likely to be extraposed than RC with lower cumulative surprisal values. We can furthermore say that we also find evidence for the second hypothesis. ID does not lose its influence over time or at least until the late nineteenth century.

## Discussion

The research presented in this paper deals with the question of why RC is in the position they are found in, i.e., adjacent or extraposed. Using a corpus of RC from the late ENHG and early NHG, we investigated the frequently mentioned factors of length and restrictiveness of RC, on the one hand, and the ID of RC, on the other hand, to find out which factors are the most influential. ID was measured in this paper in terms of cumulative surprisal values based on a skip-gram Language Model.

The results of the investigation show that both types of RC occur in all investigated time periods. Also, the ratio of extraposed to embedded RC is balanced except for the period 1750–1800.

Looking at the factors for the positioning of RC, we find strong evidence for our hypothesis that high cumulative surprisal values are the strongest predictor for extraposition. This is in contrast to previous findings on RC extraposition being prevalent in literature.

Previous research on RC agrees that for both English and German, the length of the RC is the main criterion for whether it becomes extraposed or embedded (Shannon, 1992; Uszkoreit et al., 1998; Francis and Michaelis, 2012, 2014, 2017; Levy et al., 2012). The idea deals with the fact that longer relative clauses also influence the processability of the whole sentence. If they were placed in the middle field, their integration into the rest of the sentence would cause too much processing effort, which would jeopardize the processability of the sentence (Hawkins, 1994; Gibson, 1998). Length is thus synonymous with processing effort. While we cannot refute this idea, we can show that length does not directly equate to informativeness which is also highly connected to processing efforts [Levy (2008), among others]. We have shown in section Methodological Considerations About RC Extraposition and ID that, according to the concept of information theory, the information content of a word can be lowered by inserting further material into the sentence and thereby creating a drop in individual surprisal values on individual words. It is therefore possible to prevent very high surprisal values by increasing the sentence length and thus perhaps even reduce the overall processing effort. We showed, using German RC, that the information density of a sentence is a more meaningful approach to the extraposition of RC than sentence length.

In fact, a direct comparison shows that length predicts the position of the relative clause less well than information density. We found evidence for our hypothesis in general and showed that relative clauses with high cumulative surprisal values have a higher tendency to be extraposed than relative clauses with low cumulative surprisal values.

For the two time periods from 1700 to 1750 and from 1850 to 1900, however, further argumentation is needed to corroborate the hypothesis. The period from 1700 to 1750 is the only one that does not yield a significant result for the influence of ID on extraposition. Only length is a good predictor in this model. We attribute this result to the selection of the sub-corpus and the period itself. As our corpus only includes one text, Unzer (1746), the style of writing of the author determines the results. This author mainly uses RC with low informativeness but many words. Our hitherto unpublished analysis of other, albeit theological, texts from this period shows that length is not the main factor for extraposition. It can therefore be assumed that our result is at least partly due to the selection of the corpus for this period.

The time of text publication may be a reason. The sentence frame establishes itself in the eighteenth century and the justifications for post-field setting also begin to resemble those given for modern German (Konopka, 1996). Primarily, length and informational aspects such as the setting of two emphases are mentioned again in addition to dependency-related reasons such as the avoidance of too long distances. This is especially the case for middle fields that are too long when the distance between LSB and RSB becomes too great (Konopka, 1996, p. 131). This would argue for embedding short RC. Similar recommendations are also found among late seventeenth century grammarians, so one can conclude that this developmental process may have begun during this period. Therefore, the majority of the texts available to Unzer may have had rather short middle fields without long relative clauses with little information content, which may have influenced his own writing style. Nevertheless, even this does not fully clarify the facts found. Other research also shows an influence of length in earlier and later periods, which we cannot show. Of course, this in turn may also be influenced by the text type, which remains to be verified. It must be said that it is highly probable that the deviations in the period 1700–1750 are due to a weakness in the corpus selection and that further checks are therefore necessary.

The second time period for which an influence of the ID cannot be shown in the final analysis is the last in the corpus (1850–1900). Here, we found no correlation between length and extraposition. However, the interaction between length and cumulative surprisal cannot be excluded from the model without significantly degrading it. Therefore, it cannot be clearly concluded whether the cumulative surprisal value of a relative clause or its length exerts a stronger influence on extraposition. Yet, both surprisal calculation methods (mean and cumulative surprisal) exert a marginal influence on extraposition, while length with a *p*-value of 0.97 can be ruled out as an influencing factor in the combination. The influence thus seems to definitely be present, but it cannot be completely decoupled from the length. On the one hand, this could be an indication that length does have a decisive influence on the extraposition process and that the results of, e.g., Uszkoreit et al. (1998) would be just as confirmed in studies of modern texts as those of Levy et al. (2012) among others for English. We must, however, refer to the still insufficient research situation. Whether a change is actually initiated in the late nineteenth century would become clear if the same result could be reproduced for later texts.

Apart from these two periods, our results are very clear and provide strong evidence for our first hypothesis: Extraposition and embedding are influenced by the ID of the RC.

This observation is integrated into already existing theories of information density. High information content is co-indicated with processing difficulties [Levy (2008) among others]. This approach is also intuitively understandable. If a sentence contains a lot of information, it is more strenuous to understand it. Therefore, it is important to encode the complex content in a way that keeps the processing effort as small as possible otherwise the transmitted information might be lost. In the case of the RC studied here, this is done by moving them to another position in the sentence. According to the theories of Hawkins (1994) and Gibson (1998), this results in more free cognitive capacity since the matrix sentence to the RC has already been fully processed. It should be noted here that our Language Models only pick up the lexical information of the words in the RC since they have been trained on the lemmata. Grammatical information could not be included in the consideration of the RC extraposition due to the already mentioned bad POS tagging of the DTA texts. Grammatical information could bring an additional dimension, since not only the lexical information has to be processed, but also the parts of speech behind it could be included in the consideration. For example, it has already been shown that the insertion of a function word can weaken the information content of the following content word (Jaeger, 2010).

These observations from other studies (Jaeger, 2005, 2010; Frank and Jaeger, 2008) are closely related to the UID (Levy and Jaeger, 2007). However, the UID was mainly considered in case of local changes in the information profile. The most famous example is the reduction of the optional *that* [Levy and Jaeger (2007) among others], the presence of which leads to a too low information content on the onset of the RC. Such a differentiated approach to the UID is not possible with our data. Due to different spellings and the specific subject matter of the texts, a considerable number of words is still not contained in the lexicon of the Language Model (see section Corpus and Method), so that local approaches within the RC are not possible in a meaningful way.

Previous studies on the occupation of the postfield [Speyer (2011); Sapp (2014); Coniglio and Schlachter (2015) on even older language stages of German] report a decreasing influence of information structure on the occupation of the PoF. Interaction between information structure and ID can be assumed (Speyer and Lemke, 2017) but has also not yet been studied in detail for German. If we now look at the values available here from 1650 to 1850 and exclude the time period 1700–1750, this impression could also be somewhat confirmed with regard to ID. Although ID measurements are significant or even highly significant in each case, a minimal decreasing tendency can nevertheless be detected.

There are also factors that have proven to have little or no influence. These include, contrary to the opinion of the literature, the restrictiveness of RC. For Modern German, it is assumed that restrictive RC can be better extraposed than non-restrictive RC. The reason given for this is that RC is necessary to clearly identify its antecedent. The RC is, in other words, expected because the design of the head noun makes the presence of RC highly probable. The assumed surprisal for the construction should be small, even if it occurs later in the sentence. The indication for restrictiveness does not automatically allow predictions about the content of the RC^15^. The predictions about the position of the RC made in Hypothesis 1 still carry weight and the RC is extraposed in a more unpredictable content.

In our study, the data, as a whole, shows only a marginal influence of relative clause type on extraposition (*p* < 0.1). Since, in some cases, we could not determine the type with absolute certainty, as reported in section Information Density, there is a discrepancy between the level of knowledge of the annotators and the possible world knowledge of the text authors, which often led to the type not being determined. The possibilities for error in the determination are therefore present and not negligible. Moreover, the value tends to indicate that restrictive RC is embedded, which would contradict the existing literature on the correlation between extraposition and type. Correlations between cumulative or mean surprisal and type were also not found. So, even the marginal correlation that could be found cannot be attributed to processing effort. However, the expectation regarding the relative clause could be more due to grammatical factors, as already suggested above, and less to lexical content. In fact, the arguments regarding the extraposition of restrictive RC are never about whether the content is expectable. Only the existence of the RC is described as necessary. It could, therefore, also be worth combining part-of-speech with the lexical surprisal values for this partial aspect.

Before the final summary, we will take a brief look at the second hypothesis. We proposed that ID as a principle is valid over all time steps. In fact, there is no change in its influence on the RC position, except for the period of 1700–1750 we discussed previously. At least with the help of our calculation methods, it can be concluded that information density seems to have a constant influence on the design of sentences in early NHG. Also, the presence of other styles, such as the Latin syntax, which authors of scientific articles may have been familiar with does not influence the design of German sentences in a way that should violate the principles proposed by the ID. Efficient processability of sentences is a basic principle of sentence design at all time levels. We can therefore also consider our second hypothesis as confirmed.

## Conclusion

We conclude that ID, measured as cumulative surprisal, is the best way to predict the position of a relative clause in the present corpus of medical texts from the seventeenth to nineteenth centuries. The length which was previously said to be the most influential predictor for extraposition can only present its influence in one period. This finding might be attributed to a poor choice of sub-corpus and should therefore be treated with caution. The same holds for restrictiveness. This factor does not yield significant results on the basis of this corpus. Furthermore, ID is a stable influencing factor in all time stages and can therefore be called a universal principle for the design of sentences even in earlier stages of German.

## Data Availability Statement

The annotated data set is available at https://github.com/SFB1102/C6Samples.

## Author Contributions

SV did research and writing. AS did an advisory contribution. Both authors contributed to the article and approved the submitted version.

## Funding

This paper is based on material collected in the context of the CRC, Information Density and Linguistic Encoding, funded by the Deutsche Forschungsgemeinschaft (DFG, German Research Foundation)–Project-ID 232722074–SFB 1102. Most of the material was presented at the Workshop *Rational Approaches in Language Science (RAILS) 2019*.

## Conflict of Interest

The authors declare that the research was conducted in the absence of any commercial or financial relationships that could be construed as a potential conflict of interest.

## Publisher's Note

All claims expressed in this article are solely those of the authors and do not necessarily represent those of their affiliated organizations, or those of the publisher, the editors and the reviewers. Any product that may be evaluated in this article, or claim that may be made by its manufacturer, is not guaranteed or endorsed by the publisher.

## Footnotes

^1^This way of dividing a sentence follows the “Topologisches Feldermodell” (Drach, 1937; Wöllstein, 2014), a model describing the distribution of constituents and verbs across German clauses. The verbal parts built the framework of the clause *via* the left and right sentence bracket. In main clauses, the left sentence bracket (LSB) is filled with the finite verb, whereas the right sentence bracket (RSB) holds infinite verbal parts or verbal particles. In subordinate clauses, conjunctions fill the LSB and the verb the RSB. The position in front of the LSB (Prefield) can usually be filled with one constituent and must be present to signal that a clause is a main clause. It can only be dropped in polar questions and in German dependent clauses with a complementizer, while the field between the brackets (Middlefield) can—theoretically—be filled with an arbitrary number of constituents or remain empty. The Postfield (PoF) is, if present, mostly occupied with subordinate clauses—independent and dependent clauses as in the case of RC.

^2^We use the word extraposition only as an expression which describes the separation of head noun and antecedent without referring to any generative theory for RC placement.

^3^Early New High German is commonly understood as the period of the German language spoken from 1350 to 1650. Predecessors of ENGH were Old High German (500–1,050) and Middle High German (1,050–1,350). The New High German period begins about 1700 (Nübling et al., 2013). In this study, we use the term late ENHG until 1700, following Polenz (2010).

^4^The definition of Hentschel and Weydt (2003) contrasts with the one presented in Eisenberg (1999) or Helbig and Buscha (2001). The latter two claim that subordinate conjunctions can also initiate relative clauses. We do not follow their works because, unlike relative pronouns, subordinate conjunctions can only relativize clauses to certain nouns which have a similar meaning as the conjunction itself. A temporal conjunction can, for example, only follow a noun which denotes a point in time. Relative pronouns, on the other hand, can follow any noun.

^5^In some cases, it is not easily determinable whether a RC is restrictive or not when the context or the world knowledge does not disambiguate the RC type. That will be relevant especially for the historical data because modern readers, including us as researchers, might not be able to reconstruct the knowledge of the author and his audience. The fictional example (5) shall explain this: 5) The physician treats the patient with a bezoar that is taken from a cat. A modern reader cannot say whether a bezoar—an ingredient for a remedy that is mentioned in two texts in the corpus (Purmann, 1680; Abel, 1699)—is always taken from cats or not and whether it was assumed to make a difference for the success of the treatment and is thus not able to determine the RC type.

^6^They can still have different surprisal values or contain more or less information depending on the context these variations occur in.

^7^The DTA-Project started more than ten years ago and is, therefore, not on a level we are used to in newer projects like the *Referenkorpus Frühneuhochdeutsch (ReF)*. We are aware of updates on the data but have not included possible improvements on the annotations because we manually annotated relevant information on downloaded versions of the texts. The time of the download was 2018, so this is the version of the corpus used here.

^8^As requested by one reviewer, we want to explain grammatical and lexical predictability briefly here. We understand grammatical predictability as the likelihood certain grammatical categories following each other. This could be measured using dependencies or POS-tags, e.g., to measure how likely it is for a relative pronoun to follow verbal material. This is not applicable to our current study for two reasons. First, our corpus is not dependency-parsed and has poor POS-tagging, preventing measuring grammatical predictability on our data. Second, grammatical predictability does not provide insights regarding how difficult the processing of clausal content is. This is measured using the likelihood of a certain lexical word in a context, e.g., how likely is “advice” following “medical.”

^9^These texts are part of a larger corpus of 33 texts with 593,086 tokens which includes theological texts as well, created as part of the CRC. We have collected data from these texts but processed only the mentioned nine texts so far.

^10^We must thank Katrin Ortmann (RUB) for setting up and curating WebAnno and for trying to improve the POS-tagging at this point.

^11^This might include noun-related continuous RC as well, tough their position is not variable, as pointed out by a reviewer. If their exclusion significantly changes any of the results presented in section Results: Information Density and Length, will be topic of another study.

^12^The list of the texts and the downloads of these texts can be provided on request. The same holds for R-scripts and the corpus in its current form.

^13^Position ~ (cumulative surprisal + mean surprisal + length+ restrictiveness)^2^.

^14^Position ~ (cumulative surprisal + mean surprisal + length)^2^.

^15^The determiner *derjenige* (“that one”), for example, expresses the need for a restrictive RC but does not allow any conclusions about the content of that RC, because *derjenige* is semantically neutral.
